# Cardiovascular Comorbidities and Pharmacological Treatments of COVID-19 Patients Not Requiring Hospitalization

**DOI:** 10.3390/ijerph18010102

**Published:** 2020-12-25

**Authors:** Vincenzo Russo, Gaetano Piccinocchi, Vincenzo Mandaliti, Saverio Annunziata, Giovanni Cimmino, Emilio Attena, Nicola Moio, Pierpaolo Di Micco, Sergio Severino, Roberta Trotta, Michele Del Guercio

**Affiliations:** 1 Cardiology Unit, Department of Translational Medical Sciences, University of Campania Luigi Vanvitelli—Monaldi Hospital, 80131 Naples, Italy; giovanni.cimmino@unicampania.it; 2 Comegen Primary Care Physicians Cooperative SIMG, Italian Society of Family Medicine, 80125 Naples, Italy; gpiccino@tin.it (G.P.); vincenzo.mandaliti@fastwebnet.it (V.M.); 3 KOS Primary Care Physicians Cooperative, 80128 Naples, Italy; savann.dott@gmail.com; 4 Cardiology Unit, Cotugno Hospital, 80131 Naples, Italy; emilioattena@hotmail.it (E.A.); srgsev@yahoo.it (S.S.); 5 Cardiology Department, Santa Maria delle Grazie Hospital, 80078 Pozzuoli, Italy; damosdax@libero.it; 6 Medicine Unit, Fatebenefratelli Hospital, 80131 Naples, Italy; pdimicco@libero.it; 7 Medical Affairs Department—Daiichi Sankyo, 00142 Roma, Italy; roberta.trotta@daiichi-sankyo.it; 8 Angiology Unit, District 24, Health Authority Naples 1, 80131 Naples, Italy; micheledelguercio@virgilio.it

**Keywords:** COVID-19, hypertension, cardiovascular diseases, venous thromboembolism, outpatient’s setting, risk factors, experimental drugs, low molecular weight heparin, anticoagulation

## Abstract

Introduction: The Coronavirus disease 2019 (COVID-19) outbreak is a whole Earth health emergency related to a highly pathogenic human coronavirus responsible for severe acute respiratory syndrome (SARS-CoV-2). Despite the fact that the majority of infected patients were managed in outpatient settings, little is known about the clinical characteristics of COVID-19 patients not requiring hospitalization. The aim of our study was to describe the clinical comorbidity and the pharmacological therapies of COVID-19 patients managed in outpatient settings. Materials and Methods: We performed an observational, retrospective analysis of laboratory-confirmed COVID-19 patients managed in outpatient settings in Naples, Italy between 9 March and 1 May 2020. Data were sourced from the prospectively maintained Health Search (HS)/Thales database, shared by 128 primary care physicians (PCPs) in Naples, Italy. The clinical features and pharmacological therapies of COVID-19 patients not requiring hospitalization and managed in outpatient settings have been described. Results: A total of 351 laboratory-confirmed COVID-19 patients (mean age 54 ± 17 years; 193 males) with outpatient management were evaluated. Hypertension was the most prevalent comorbidity (35%). The distribution of cardiovascular comorbidities showed no gender-related differences. A total of 201 patients (57.3%) were treated with at least one experimental drug for COVID-19. Azithromycin, alone (42.78%) or in combination (27.44%), was the most widely used experimental anti-COVID drug in outpatient settings. Low Molecular Weight Heparin and Cortisone were prescribed in 24.87% and 19.4% of the study population, respectively. At multivariate regression model, diabetes (risk ratio (RR): 3.74; 95% CI 1.05 to 13.34; *p* = 0.04) and hypertension (RR: 1.69; 95% CI 1.05 to 2.7; *p* = 0.03) were significantly associated with the experimental anti-COVID drug administration. Moreover, only diabetes (RR: 2.43; 95% CI 1.01 to 5.8; *p* = 0.03) was significantly associated with heparin administration. Conclusions: Our data show a high prevalence of hypertension, more likely treated with renin–angiotensin–aldosterone system (RASS) inhibitors, among COVID-19 patients not requiring hospitalization. Experimental COVID-19 therapies have been prescribed to COVID-19 patients considered at risk for increased venous thromboembolism based on concomitant comorbidities, in particular diabetes and hypertension.

## 1. Introduction

Human coronaviruses (HCoVs) are enveloped nonsegmented positive-strand RNA viruses, with rapid evolution owing to their high genomic nucleotide substitution rates and recombination. HCoVs are associated with multiple respiratory diseases of varying severity, including common cold, pneumonia and bronchiolitis. The Coronavirus disease 2019 (COVID-19) outbreak is a whole Earth health emergency related to a highly pathogenic human coronavirus responsible for severe acute respiratory syndrome (SARS-CoV-2) [1]. In about 15% of COVID-19 patients, the clinical course of the disease may be complicated by the onset of severe interstitial pneumonia, respiratory distress syndrome (ARDS) and/or multi organ failure (MOF) that may require hospitalization; however, many infected patients remain asymptomatic or paucisymptomatic and are managed in outpatient settings.

Previous observational studies from different countries described the baseline clinical characteristics of COVID-19 patients. In particular, it emerged that SARS-CoV-2 was more likely to affect older men with cardiovascular comorbidities, such as hypertension, diabetes and coronary artery disease [2]; however, they included only hospitalized subjects admitted or not to an intensive care unit (ICU). Italy is one of the hardest-hit countries by COVID-19, with more than 236,000 laboratory-confirmed cases by 14 June 2020 [3]. During the outbreak, the Italian health authorities recommended outpatient management of patients with suspected or confirmed COVID-19 who presented with mild to moderate symptoms. Despite the majority of infected patients being managed in outpatient settings, little is known about the clinical characteristics of COVID-19 patients not requiring hospitalization. The aim of our study was to describe the cardiovascular comorbidities and the pharmacological therapies of COVID-19 patients not requiring hospitalization and managed in outpatient settings in Naples, the third most populous city in Italy.

## 2. Materials and Methods

### 2.1. Study Design and Population

We performed an observational retrospective analysis of laboratory-confirmed COVID-19 patients not requiring hospitalization and managed in outpatient settings in Naples, Italy between 9 March and 1 May 2020. Data were sourced from the prospectively maintained Health Search (HS)/Thales database, shared by 128 primary care physicians (PCPs) in Naples, Italy, which included data from the electronic patient records of all patients followed by the PCPs. PCPs voluntarily agreed to collect patient information and to attend specific training courses on data entry. The detection of SARS-CoV-2 by real-time quantitative reverse-transcription polymerase chain reaction (RT-PCR) assay on nose/throat swab or sputum sample was used to achieve the laboratory confirmation of COVID-19.

### 2.2. Data Collected

Patient demographics, cardiovascular risk factors, comorbid medical conditions, pharmacological treatments and experimental drugs used for COVID-19 were collected. Patients were excluded from the analysis if they were missing demographic information (*n* = 7), if data did not pull correctly from the electronic health record (*n* = 3) or if they had a prior hospitalization for COVID-19 (*n* = 5).

### 2.3. Objectives

Our primary endpoint was to describe the cardiovascular comorbidities and pharmacological treatment of COVID-19 patients not requiring hospitalization and managed in outpatient settings. The secondary endpoint was to evaluate the association between experimental pharmacological treatments prescribed for COVID-19 and the clinical features of the study population.

### 2.4. Statistical Analysis

Due to the observational nature of the study, a formal calculation of the study sample size was not applicable and only descriptive analyses were performed. Kolmogorov–Smirnov and the Shapiro–Wilk tests were used to evaluate the distribution of continuous data. Normally distributed variables are expressed as mean ± standard deviation (SD), whereas nonnormal distributed ones are expressed as median and interquartile range (IQR). Categorical variables are reported as numbers and percentages. For all tests, a *p*-value < 0.05 was considered statistically significant. A logistic regression model was used to calculate the adjusted risk ratios (RR) for the outcome of interest; the results were presented as RR with their 95% confidence intervals (CI). Analyses were performed by using R version 3.5.1 (R Foundation for Statistical Computing, Vienna, Austria).

## 3. Results

From the HS/Thales database, 366 laboratory-confirmed COVID-19 patients with outpatient management were selected. Patients with missing demographic information (*n* = 7), or with data that did not pull correctly from the electronic health record (*n* = 3) or with a prior hospitalization for COVID-19 (*n* = 5) were excluded. Finally, 351 laboratory-confirmed COVID-19 patients not requiring hospitalization and managed in outpatient settings were analyzed. All patients presented with fever or feeling feverish, chills and cough. The mean duration of symptoms was 26 ± 11 days. The mean age was 54 ± 17 years, and 193 patients were male. Baseline characteristics of the study population are summarized in Table 1.

Hypertension was the most prevalent comorbidity (35%), followed by chronic obstructive pulmonary disease (23.9%), dyslipidemia (12.5%) and diabetes mellitus (9.1%). The prevalence of coronary artery disease (CAD), chronic kidney disease (CKD) and atrial fibrillation (AF) was less than 4% among the study population. The distribution of cardiovascular comorbidities showed no gender-related differences; a trend in increase in CAD prevalence in male patients (5.69% vs. 1.89%; *p* = 0.0704) was found.

Angiotensin-converting enzyme inhibitors (ACEIs) and angiotensin II receptor blockers (ARBs) were the most prevalent pharmacological treatments (26.78%), followed by beta-blockers (16.80%), statins (16.52%) and antiplatelet drugs (13.39%). No gender-related difference was found.

Among the study population, 201 patients (57.3%) were treated with at least one experimental drug for COVID-19. Treated patients showed more likely history of AF (4.97% vs. 0.66%, *p* = 0.02), dyslipidemia (16.41% vs. 7.33%; *p* = 0.01), hypertension (41.79% vs. 26%; *p* = 0.002), diabetes (13.43% vs. 3.33%; *p* = 0.001), CAD (5.97% vs. 1.33%; *p* = 0.02) and CKD (4.97% vs. 1.33%; *p* = 0.06) compared with those not treated. The prevalence of ACEs/ARBs (34.82% vs. 16%; *p* = 0.0001); Ca-antagonists (13.93% vs. 6.66%; *p* = 0.03), Beta-blockers (20.39 vs. 12%; *p* = 0.03), antiplatelet drugs (18.90% vs. 6%; *p* = 0.0005), statins (22.38% vs. 8.66%; *p* = 0.0006), oral anticoagulant (5.97% vs. 0%, *p* = 0.002) and oral hypoglycemic agents (9.95% vs. 1.33%; *p* = 0.001) was higher in patients treated with at least one experimental drug for COVID-19 compared with those not treated (Table 2).

Among prescribed pharmacological experimental drugs for COVID-19, Azithromycin, alone (42.78%) or in combination with Hydroxychloroquine (HCQ) (10.94%), Cortisone (10.44%) or both (6.46%), was the most widely used drug in outpatients’ setting. Both Cortisone and Hydroxychloroquine alone were prescribed in a small percentage of patients, 2.48% and 1.99%, respectively. Table 3 shows all pharmacological experimental therapy used in the study population.

Low molecular weight heparin (LMWH) therapy, in particular enoxaparin 4.000 UI daily, was prescribed in 50 patients (24.87%), who showed more likely higher prevalence of hypertension (48% vs. 33%; *p* = 0.039), diabetes (18% vs. 7.64; *p* = 0.019) and CAD (10% vs. 3%, *p* = 0.02) as comorbidities (Table 4) and ACEs/ARBs (42% vs. 24.2%, *p* = 0.008), antiplatelet drugs (26% vs. 11.29%; *p* = 0.010) and oral hypoglycemic agents (18% vs. 4.31%; *p* = 0.0002) as pharmacological treatments. Moreover, COVID-19 patients on LMWH therapy were more likely treated with Azithromycin (88% vs. 46.84%; *p* < 0.0001), Hydroxychloroquine (58% vs. 12.62%; *p* < 0.0001) and Cortisone (36% vs. 12.95%; *p* < 0.0001) compared with those not treated with LWMH. At multivariate regression model, diabetes (RR: 3.74; 95% CI 1.05 to 13.34; *p* = 0.04) and hypertension (RR: 1.69; 95% CI 1.05 to 2.7; *p* = 0.03) were significantly associated with the experimental anti-COVID drug administration. Moreover, only diabetes (RR: 2.43; 95% CI 1.01 to 5.8; *p* = 0.03) was significantly associated with LMWH administration.

## 4. Discussions

The epidemiological association between cardiovascular diseases or risk factors and COVID-19 has been previously demonstrated by many studies describing the clinical features of patients with SARS-CoV-2 infection. In particular, COVID-19 patients were more likely older men with hypertension, diabetes and coronary artery disease; this clinical phenotype seems to be similar among cohorts from different countries [2]. Actually, the data that emerged from published studies included only hospitalized subjects admitted or not to an intensive care unit.

Following the outbreak of COVID-19, from 10th March to 4th May 2020 the Italian government, in an attempt to contain the virus diffusion, adopted strict rules characterized by lockdown and social distancing [4]. In this context, the Italian health authorities recommended outpatient management of patients with suspected or confirmed COVID-19 who presented with mild to moderate symptoms. Despite the majority of infected patients being managed in outpatient settings, little is known about the clinical characteristics of COVID-19 patients not requiring hospitalization.

The aim of our study was to describe the cardiovascular comorbidities and the pharmacological therapies of COVID-19 patients not requiring hospitalization and managed in outpatient settings in Naples, the third-most-populous city of Italy. The present is the first Italian observational study including COVID-19 patients not requiring hospitalization and managed in outpatient settings.

Among our study population, hypertension was the most prevalent cardiovascular comorbidity and it was more likely treated with renin–angiotensin aldosterone system (RAAS) inhibitors, regardless of gender differences. Early reports from major COVID-19 epicenters revealed higher rates of hypertension among critically ill hospitalized patients, suggesting a causal relationship between hypertension, the use of RAAS inhibitors and the severity of the disease [5,6,7]. However, studies were underway to explore whether hypertension is an independent risk factor for COVID-19, and it is not recommended to discontinue the use of RAAS inhibitors where clinically indicated [8]. Recently, the results of the Angiotensin Receptor Blockers and Angiotensin-converting Enzyme Inhibitors and Adverse Outcomes in Patients with COVID19 (BRACE-CORONA) study did not show clinical benefit, in terms of 30-day mortality rates, from routinely interrupting ACE inhibitors/ARBs in hospitalized patients with mild to moderate COVID-19 [9].

Our results suggest the hypothesis that the high prevalence of both hypertension (about 35%) and RAAS inhibitors use (about 26%) among COVID-19 patients was related to patients’ age and not to the severity of the disease; indeed, according to the Italian National Institute of Health, 31% of the Italian population between 35 and 74 years of age was hypertensive, and an additional 17% were borderline [10]. Moreover, the use of RAAS inhibitors was about 45% among Italian patients with hypertension [11].

A low prevalence of coexisting cardiovascular comorbidities, such as coronary artery disease, chronic kidney disease and atrial fibrillation, accounting for less than 4%, has been reported among COVID-19 patients not requiring hospitalization, which is different than those reported by cohort studies in hospital settings (ranging from 5 to 17%) [2,12,13]. This finding might be explained by the recommendation to refer COVID-19 patients with a low ischemic threshold related to underlying cardiovascular disease, who were at increased risk to develop myocardial injury or a clinically severe form of the disease and death [14], to emergency departments. In this clinical setting, the pathogenesis of myocardial injury may be related to SARS-CoV-2 direct myocardial damage [15] and to the imbalance between oxygen supply and demand due to respiratory failure and systemic arterial hypotension [16].

Our results confirm and integrate those from a recent American study including 208 adults with COVID-19 not requiring hospitalization and managed in outpatient settings in the state of Georgia [17]. In their retrospective evaluation, Bergquist et al. showed that 87.5% of the outpatients’ study population were younger than 65 years old and 30.3% did not have an underlying medical condition. Based on these data, the authors hypothesized that the risk of a severe form of COVID-19 may be higher in medically vulnerable older people with underlying medical conditions. The pre-admission treatment with antithrombotic drugs did not seem to show a protective effect in more severe forms of COVID-19 characterized by ARDS and rapidly evolving toward death [18].

Among our study population, the experimental COVID-19 treatments in outpatient settings were used in more than half of the study population (57.3%) and Azithromycin, alone (42.78%) or in combination with Hydroxychloroquine (10.4%) or Cortisone (10.94%), was the most widely used drug. The high prevalence of Azithromycin use in outpatient settings might be explained by early data supporting its potential effectiveness in SARS-CoV-2 infection [19], the PCPs prescribing with confidence due to the wide use of Azithromycin for skin, respiratory and other infections, and because it is generally considered a safe medication. In contrast, the low prevalence of Hydroxychloroquine use among our study population may be explained by the insufficient and often conflicting evidence on the benefit and harms of using HCQ to treat COVID-19 [20], the low prescribing PCPs confidence and the risk of QT prolongation [21].

As worldwide cohort studies suggested a high rate of thromboembolic complications among hospitalized COVID-19 patients [22], the anticoagulant thromboprophylaxis with LMWH, low dose unfractionated heparin twice daily, or fondaparinux has been recommended for acutely ill hospitalized medical patients at increased risk of thrombosis [23]. Moreover, the use of anticoagulant therapy with heparin has been shown to decrease mortality in hospitalized patients with severe COVID-19 [24]. LMWH or fondaparinux should be preferred over unfractionated heparin due to the decreased risk of bleeding, good predictability, dose-dependent plasma levels and longer half-life [25]. Among our study population, the experimental COVID-19 treatments, alone or in combination, were prescribed in COVID-19 patients who showed more likely concomitant comorbidities such as diabetes mellitus, hypertension and CAD; and, among them, only diabetes and hypertension have been independently associated with the administration of at least one experimental COVID-19 drug in outpatient settings. This data may be explained by the PCPs’ awareness that diabetes and hypertension cause endothelial dysfunction or vascular inflammation [26] and predispose to increased risk of venous thromboembolism [27].

### Limitation

Despite the novelty, our study is limited by the retrospective observation nature, relatively small sample size and restricted geographical location. Another limit may be represented by the methods of data collection, including electronic patient record information from the Health Search (HS)/Thales database. However, it represents the first report in the literature about the cardiovascular comorbidities and pharmacological characteristics of COVID-19 patients not requiring hospitalization and managed in outpatient settings.

## 5. Conclusions

Our data show a high prevalence of hypertension, more likely treated with RAAS inhibitors, among COVID-19 patients not requiring hospitalization and managed in outpatient settings. This finding denies the hypothesis of a causal association between hypertension and hypertensive treatments with critically ill COVID-19 patients. Moreover, the low prevalence of coexisting cardiovascular comorbidities among our study population suggests that clinical features of COVID-19 adults managed in outpatient settings differ from those requiring hospitalization. Additionally, experimental COVID-19 therapies have been prescribed to COVID-19 patients considered at increased risk for venous thromboembolism based on concomitant comorbidities, in particular diabetes and hypertension. Azithromycin, LWMH and Cortisone were prescribed in 70.62%, 24.87% and 19.4% of the study population, respectively.

## Figures and Tables

**Table 1:** Patients’ characteristics and sex distribution among the overall COVID-19 study population.

Variable	Overall	Male	Female	*p-*Value
	n: 351	n: 193	n: 158	
Age (mean ± SD)	53.52 ± 17.17	53.52 ± 17.17	53.46 ± 17.23	0.999
Smokers, *n* (%)	19 (5.41%)	15 (7.77%)	4 (2.53%)	0.0311
AF, *n* (%)	11 (3.13%)	7 (3.62%)	4 (2.53%)	0.5601
Dyslipidemia, *n* (%)	44 (12.53%)	28 (14.50%)	16 (10.12%)	0.2181
Hypertension, *n* (%)	123 (35.04%)	69 (35.75%)	54 (34.17%)	0.7579
Diabetes Mellitus, *n* (%)	32 (9.11%)	19 (9.84%)	13 (8.22%)	0.6003
CAD, *n* (%)	14 (3.98%)	11 (5.69%)	3 (1.89%)	0.0704
CKD, *n* (%)	12 (3.41%)	6 (3.10%)	6 (3.79%)	0.7235
COPD, *n* (%)	84 (23.93%)	41 (21.24%)	43 (27.21%)	0.1928
ACEIs/ARBs, *n* (%)	94 (26.78%)	55 (28.49%)	39 (24.68%)	0.4232
Ca-antagonists, *n* (%)	38 (10.82%)	22 (11.39%)	16 (10.12%)	0.7035
Beta-blockers, *n* (%)	59 (16.80%)	33 (17.09%)	26 (16.45%)	0.8734
Amiodarone, *n* (%)	1 (0.28%)	0	1 (0.63%)	0.2702
Antiplatelet drugs, *n* (%)	47 (13.39%)	30 (15.54%)	17 (10.75%)	0.1904
Statins, *n* (%)	58 (16.52%)	39 (20.20%)	19 (12.02%)	0.0403
Anticoagulants, *n* (%)	12 (3.41%)	6 (3.10%)	6 (3.79%)	0.7235
Insulin, *n* (%)	12 (3.41%)	6 (3.10%)	6 (3.79%)	0.7235
Oral hypoglycemic agents, *n* (%)	22 (6.26%)	15 (7.77%)	7 (4.43%)	0.1996
Ivabradine, *n* (%)	4 (1.13%)	2 (1.03%)	2 (1.26%)	0.8397

AF, atrial fibrillation; CAD, coronary artery disease; CKD, chronic kidney disease; COPD, chronic obstructive pulmonary disease, DCM, dilated cardiomyopathy; ACEI, angiotensin-converting enzyme inhibitor; ARBs, angiotensin II receptor blockers.

**Table 2:** Clinical features of patients treated with at least one experimental drug for COVID-19 versus not-treated patients.

	Treated Group	Non-Treated Group	*p*-Value
	n: 201	n: 150	
Age (mean ± SD)	53.38 ± 17.19	53.24 ± 17.22	0.999
Male, *n* (%)	115 (57.21%)	78 (52%)	0.33
Smokers, *n* (%)	12 (5.97%)	7 (4.66%)	0.59
AF, *n* (%)	10 (4.97%)	1 (0.66%)	0.02
Dyslipidemia, *n* (%)	33 (16.41%)	11 (7.33%)	0.01
Hypertension, *n* (%)	84 (41.79%)	39 (26%)	0.002
Diabetes Mellitus, *n* (%)	27 (13.43%)	5 (3.33%)	0.001
CAD, *n* (%)	12 (5.97%)	2 (1.33%)	0.02
CKD, *n* (%)	10 (4.97%)	2 (1.33%)	0.06
COPD, *n* (%)	51 (25.37%)	33 (22%)	0.46
ACEIs/ARBs, *n* (%)	70 (34.82%)	24 (16%)	0.0001
Ca-antagonists, *n* (%)	28 (13.93%)	10 (6.66%)	0.03
Beta-blockers, *n* (%)	41 (20.39%)	18 (12%)	0.03
Amiodarone, *n* (%)	1 (0.49%)	0	0.39
Antiplatelet drugs, *n* (%)	38 (18.90%)	9 (6%)	0.0005
Statins, *n* (%)	45 (22.38%)	13 (8.66%)	0.0006
Oral anticoagulants, *n* (%)	12 (5.97%)	0	0.002
Insulin, *n* (%)	10 (4.97%)	2 (1.33%)	0.06
Oral hypoglycemic agents, *n* (%)	20 (9.95%)	2 (1.33%)	0.001
Ivabradine, *n* (%)	4 (1.99%)	0	0.08

AF, atrial fibrillation; CAD, coronary artery disease; CKD, chronic kidney disease; COPD, chronic obstructive pulmonary disease, DCM, dilated cardiomyopathy; ACEI, angiotensin-converting enzyme inhibitor; ARBs, angiotensin II receptor blockers.

**Table 3:** Pharmacological experimental drugs for COVID-19 among treated outpatients.

	Overall	Male	Female	*p*-Value
	n: 201	n: 115	n: 86	
Azithromycin alone, (%)	86 (42.78%)	47 (40.86%)	39 (45.34%)	0.52
Hydroxychloroquine alone, *n* (%)	4 (1.99%)	3 (2.60%)	1 (1.16%)	0.47
Cortisone alone, *n* (%)	5 (2.48%)	2 (1.74%)	3 (3.48%)	0.43
Azithromycin + Hydroxychloroquine, *n* (%)	22 (10.94%)	13 (11.30%)	9 (10.46%)	0.85
Azithromycin + Cortisone, *n* (%)	21 (10.44%)	14 (12.17%)	7 (8.14%)	0.35
Azithromycin + Hydroxychloroquine + Cortisone, *n* (%)	13 (6.46%)	7 (6.08%)	6 (6.97%)	0.80
Low Molecular Weight Heparin, *n* (%)	50 (24.87%)	29 (25.21%)	21 (24.41%)	0.89

**Table 4:** Clinical features of COVID-19 patients with and without low molecular weight heparin (LWMH).

	LWMH Group	No LWMH Group	*p*-Value
	n: 50	n: 301	
Age (mean ± SD)	53.48 ± 17.37	53.46 ± 17.15	0.999
Male, *n* (%)	29 (58%)	164 (54.5%)	0.645
Smokers, *n* (%)	4 (8%)	15 (5%)	0.386
AF, *n* (%)	1 (2%)	10 (3.32%)	0.620
Dyslipidemia, *n* (%)	5 (10%)	39 (13%)	0.554
Hypertension, *n* (%)	24 (48%)	99 (33%)	0.039
Diabetes Mellitus, *n* (%)	9 (18%)	23 (7.64%)	0.019
CAD, *n* (%)	5 (10%)	9 (3%)	0.02
CKD, *n* (%)	2 (4%)	10 (3.32%)	0.806
COPD, *n* (%)	8 (16%)	76 (25.2%)	0.158
ACEIs/ARBs, *n* (%)	21 (42%)	73 (24.2%)	0.008
Ca-antagonists, *n* (%)	6 (12%)	32 (10.63%)	0.773
Beta-blockers, *n* (%)	10 (20%)	49 (16.27%)	0.514
Amiodarone, *n* (%)	0	1 (0.33%)	0.684
Antiplatelet drugs, *n* (%)	13 (26%)	34 (11.29%)	0.010
Statins, *n* (%)	10 (20%)	48 (15.94%)	0.474
Oral anticoagulants, *n* (%)	3 (6%)	9 (2.99%)	0.278
Insulin, *n* (%)	3 (6%)	9 (2.99%)	0.278
Oral hypoglycemic agents, *n* (%)	9 (18%)	13 (4.31%)	0.0002
Ivabradine, *n* (%)	1 (2%)	3 (0.99%)	0.532
Azithromycin, *n* (%)	44 (88%)	141 (46.84%)	<0.0001
Hydroxychloroquine, *n* (%)	29 (58%)	38 (12.62%)	<0.0001
Cortisone, *n* (%)	18 (36%)	39 (12.95%)	<0.0001

AF, atrial fibrillation; CAD, coronary artery disease; CKD, chronic kidney disease; COPD, chronic obstructive pulmonary disease, DCM, dilated cardiomyopathy; ACEI, angiotensin-converting enzyme inhibitor; ARBs, angiotensin II receptor blockers.

## Data Availability

The data presented in this study are available on request from the corresponding author.
